# The effect of acute pomegranate extract supplementation on oxygen uptake in highly-trained cyclists during high-intensity exercise in a high altitude environment

**DOI:** 10.1186/s12970-017-0172-0

**Published:** 2017-05-31

**Authors:** Emma May Crum, Ahmad Munir Che Muhamed, Matthew Barnes, Stephen Robert Stannard

**Affiliations:** 1 School of Sport and Exercise, Massey University (New Zealand), Palmerston North, New Zealand; 2 Advanced Medical and Dental Institute, Universiti Sains Malaysia, George Town, Malaysia

**Keywords:** Nitrates, Polyphenols, Ergogenic aids, Exercise performance

## Abstract

**Background:**

Recent research has indicated that pomegranate extract (POMx) may improve performance during aerobic exercise by enhancing the matching of vascular oxygen (O_2_) provision to muscular requirements. POMx is rich in ellagitannin polyphenols and nitrates (NO_3_^−^), which are both associated with improvements in blood flow and O_2_ delivery. Primarily, this study aimed to determine whether POMx improves performance in a cycling time trial to exhaustion at 100%VO_2max_ (TTE100%) in highly-trained cyclists. In addition, we investigated if the O_2_ cost (VO_2_) of submaximal exercise was lower with POMx, and whether any changes were greater at high altitude where O_2_ delivery is impaired.

**Methods:**

Eight cyclists exercised at three submaximal intensities before completing a TTE100% at sea-level (SEA) and at 1657 m of altitude (ALT), with pre-exercise consumption of 1000 mg of POMx or a placebo (PLAC) in a randomized, double-blind, crossover design. Data were analysed using a three way (treatment x altitude x intensity) or two-way (treatment x altitude) repeated measures ANOVA with a Fisher’s LSD post-hoc analysis. Significance was set at *p* ≤ 0.05. The effect size of significant interactions was calculated using Cohen’s d.

**Results:**

TTE100% performance was reduced in ALT but was not influenced by POMx (*p* > 0.05). Plasma NO_3_^−^ were 10.3 μmol greater with POMx vs. PLAC (95% CI, 0.8, 19.7,*F*_1,7_ = 7.83, *p* < 0.04). VO_2_ measured at five minutes into the TTE100% was significantly increased in ALTPOMx vs. ALTPLAC (+3.8 ml.min^−1^kg^−1^, 95% CI, −5.7, 9.5, F_1,7_ = 29.2, *p* = 0.001, ES = 0.6) but unchanged in SEAPOMx vs. SEAPLAC (*p* > 0.05). Submaximal VO_2_ values were not affected by POMx (*p* ≥ 0.05).

**Conclusions:**

The restoration of SEA VO_2_ values at ALT is likely driven by the high polyphenol content of POMx, which is proposed to improve nitric oxide bioavailability. Despite an increase in VO_2_, no change in exercise performance occurred and therefore this study does not support the use of POMx as an ergogenic supplement.

## Background

Pomegranate (*Punica granatum*) (POM), is a seeded, red, fleshy fruit of Middle East origin which was used in traditional medicine to treat a variety of inflammatory conditions [1]. In modern-day research, the health benefits of POM have been attributed to its high concentration of nitrates (NO_3_^−^) and polyphenol compounds, and consumption of POM juice (POMJ) or extract (POMx) has been linked to a decline in cancer proliferation [2], the amelioration of cardiovascular disease markers [3] and decreases in gut and joint inflammation [4, 5]. Recent research has indicated that POM-based supplements can also improve performance during aerobic exercise by enhancing the matching of vascular O_2_ provision to muscular requirements [6, 7].

Polyphenols are a group of phytochemicals with antioxidant properties that contain one or more aromatic rings and at least two hydroxyl groups [8]. POMJ contains a greater concentration of polyphenols (~3.8 mg.ml^−1^) than other polyphenol-rich beverages such as red wine (~3.5 mg.ml^−1^), Concord grape juice (~2.6 mg.ml^−1^) and cranberry juice (~1.7 mg.ml^−1^) [9]. These are predominantly from the ellagitannin (ET) subclass (80–90%) with smaller amounts of anthocyanins (8–15%) [10]. Consumption of ET or anthocyanin-rich foods is associated with a decrease in systolic blood pressure (SBP) and an increase in vessel diameter and blood flow [6, 11, 12] suggesting a link between POM consumption and O_2_ delivery.

Supplementation with dietary NO_3_^−^ influences O_2_ delivery during exercise through its conversion to the potent vasodilator, nitric oxide (NO). During resting conditions, NO is primarily produced endogenously by NO synthases (NOS) [13]. However, in hypoxic conditions, such as those present locally in the muscles during exercise, activity of this pathway is limited, placing greater importance on the secondary NO_3_^−^-nitrite (NO_2_^−^)-NO pathway [14]. Polyphenols further enhance the effects of dietary NO_3_^−^ by promoting their conversion into NO [15, 16], and protecting NO from damage caused by reactive oxygen species (ROS) [17].

Previous research involving NO_3_^−^ supplementation has predominantly used beetroot juice (BRJ), which contains ~11 mmol.L^−1^ NO_3_^−^ [18] in comparison to 12.93 ppm.L^−1^ (~0.2 mmol.L^−1^) in POMJ and 109 ppm.L^−1^ (~1.76 mmol.L^−1^) in POMx, as reported by Roelofs et al. [6]. Acute or short-term BRJ supplementation in low to moderately-trained individuals is associated with a large increase (>92%) in plasma NO_2_^−^, a 3–5% reduction in O_2_ uptake (VO_2_) during submaximal exercise and a 15–25% improvement in performance during cycling, running and knee extension time to exhaustion protocols [18–22]. In contrast, BRJ has little or no benefit on these parameters in highly-trained athletes (VO_2max_ >60 ml.min^−1^kg^−1^), who may have greater NOS activity and consequently a lower reliance on NO production via NO_3_^−^ [23–29]. However, BRJ appears to benefit these athletes during exercise at high altitude, where the lower atmospheric pressure of O_2_ (PO_2_) impairs O_2_ transport and places greater reliance on the non-aerobic pathway to produce NO [30, 31]. This is significant because exercise at high altitude features in a number of major sporting competitions, such as the Tour de France.

While BRJ clearly has an effect on VO_2_, the high concentration of ET in POM may provide a source of dietary NO_3_^−^ with greater bioavailability, despite the lower total NO_3_^−^ content. Currently, only two studies have investigated the effect of POM supplementation on endurance exercise performance at sea-level, with no previous research investigating its effects in low PO_2_ conditions. In moderately-trained individuals, acute POMx intake (1000 mg) increased pre-exercise blood flow and time to exhaustion during treadmill running at 90-100% of peak velocity [7]. Conversely, following seven days of POMJ supplementation (1000 ml.day^−1^) in a trained cohort, no change in performance was observed during a ten minute cycling time trial or time to exhaustion protocol in hot conditions [32]. Thus, further research is warranted to determine whether POM improves O_2_ transport and endurance exercise performance.

The outcomes of the current study are primarily to further explore the effect of acute POMx supplementation on endurance exercise performance, and secondarily, to determine the effect of POMx on O_2_ transport parameters. In addition, the study will investigate whether any observed effects are greater during exercise in a high altitude environment. We hypothesize that acute POMx supplementation will reduce the submaximal O_2_ cost of exercise, and in doing so, improve performance in an environment (altitude) where O_2_ availability may be limiting.

## Methods

### Participants

All participants provided written consent after being informed about the study requirements and benefits and risks of participating. A health questionnaire was also completed. Eight highly-trained cyclists, including seven males and one female, were recruited from the regional cycle community. This sample size was chosen based on previous studies which have successfully shown a decrease in VO_2_ following NO_3_^−^ supplementation with a sample size of 8 participants [18, 21]. While the sample size is small, this was unavoidable given the highly-trained status of participants that we wished to study. All participants were current or past members of the national cycling or triathlon junior development programmes. Their age, height, body mass and peak aerobic capacity (VO_2max_) were 17–18 years, 67.6 ± 7 kg, 180 ± 9 cm and 74.4 ± 6.2 ml.min^−1^kg^−1^ respectively. The study was approved by the Massey University Human Ethics Committee (Southern A 15/54) in accordance with the Declaration of Helsinki.

### Experimental design

#### VO_2max_ test

In order to determine workloads for the supplemented trials, participants completed a VO_2max_ test in thermoneutral conditions (18–20 °C) at sea-level, on an electronically-braked cycle ergometer (Lode Excalibur Sport, Groningen, The Netherlands), which was set up as closely as possible to the participant’s own bike. This session doubled as an initial familiarization session to ensure participants were familiar with the equipment and protocols involved in the experimental sessions. Following a five minute warm-up at 100 W, participants completed four × 7-min stages of increasing workload (e.g., 150, 200, 250, 300 W) with expired air being collected into Douglas Bags during the last minute of each stage and analysed for O_2_ and CO_2_ concentrations and volume. Following a five minute active rest period, an incremental “ramp” protocol was used to determine VO_2max_. Power began at 100 W and increased linearly with time (25 W.min^−1^). Participants cycled for as long as possible and verbal encouragement was given to elicit maximal effort. As the participant’s VO_2max_ was approached (as indicated by a change in breathing pattern), expired air was captured in Douglas bags until exhaustion. Analysis of Douglas bags was done using a calibrated gas analysing system (AD Instruments, Dunedin, New Zealand). The gas analyser was calibrated using gases of known concentration (15.01% O_2_, 5.01% CO_2_). Minute ventilation (V_E_) and concentrations of O_2_ and CO_2_ values were used to calculate the volume of inspired air (V_I_) using the Haldane transformation, where V_E_ was corrected for barometric pressure, ambient temperature and atmospheric water saturation. Subsequently, VO_2_ and expired CO_2_ (VCO_2_) could be determined and are reported as standard temperature and pressure dry (STPD). The respiratory exchange ratio (RER) was calculated using VCO_2_/VO_2_ and attainment of VO_2max_ was confirmed with RER ≥1.1. A relationship between steady-state workload and VO_2_ values was drawn through creation of a power curve and generation of a linear line equation y = mx + c, where m = gradient, x = power and c = start point. The equation was used to estimate power output at 50, 65 and 80% of VO_2max._

#### Experimental protocol

**Fig. 1:**
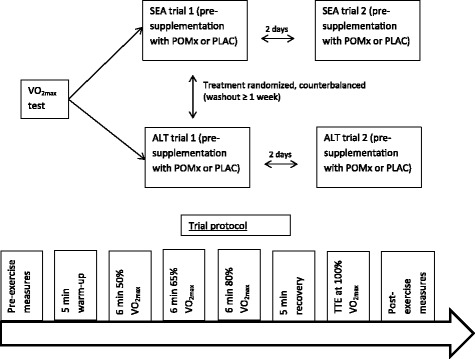
Schematic representation of the study design

The experimental protocol (Fig. 1) was a randomized, double-blind, crossover design, which was completed on four occasions: twice in sea-level conditions (SEA) and twice in high-altitude conditions (ALT, 1657 m, ~17% O_2_). The testing location was the Turoa ski-field carpark, Ohakune, which was chosen due to being the highest road accessible to our mobile laboratory in the North Island of New Zealand. Prior to each trial, participants ingested 1000 mg of either a POMx supplement (POM Wonderful LLC, USA) in capsule form or a placebo capsule of the same colour, size and shape as POMx (PLAC, brown sugar). The quantity of POMx was based on Trexler et al. [7] who showed an association between acute POMx supplementation and improved running performance. Previous analysis of the POM Wonderful LLC supplement have shown it to contain 1800 ppm polyphenols, comprised of 95.5% ellagitannins, 3.5% ellagic acid and 1% anthocyanins [32]. Participants completed a POMx trial and a PLAC trial in each environmental condition in a randomized order; the supplement blinded to the participant and the researcher. The participant was instructed to swallow the capsule whole, without tasting it, to avoid an expectation bias. In accordance with previous POMx research, the supplement was ingested 2.5 h prior to each experimental trial to allow maximal absorption prior to exercise [9]. During the 48 h prior to each session, participants were asked to limit consumption of NO_3_^−^ and polyphenol-rich foods and avoid strenuous exercise and antibacterial substances, such as mouthwash, which destroy NO_3_^−^ -NO_2_^−^ converting bacteria on the tongue [33]. Participants consumed a standardized meal (524 cal, 8 g protein, 11 g fat, 51 g carbohydrate) three hours prior to exercise. Participants were asked to arrive at the testing session in a hydrated state, and consumed water ad libitum throughout the trial.

On arrival to the laboratory, body mass was obtained and a heart rate (HR) monitor (Garmin, Kansas, USA) was applied and recorded HR at a sampling rate of two seconds throughout the trial. After sitting in a supine position for five minutes, blood pressure of the brachial artery was measured using an automated sphygmomanometer (Japan Precision Instruments, Gumma, Japan). Blood was collected via fingerprick sample (~5 μL) and analysed for blood lactate concentration ([La^−^]_b_) using a blood lactate test meter (Lactate Pro, Arkray KDK, Japan). To determine haematocrit (Hct), additional blood (~35 μL) was collected from the fingertips in heparinized capillary microtubes and immediately spun in a microhaematocrit centifuge (Thermo IEC MB, Bellport, USA) at 14,000 rpm for two minutes. Hct was calculated as the length of red blood cells as a percentage of the total length of blood in the tube. Samples were taken in duplicate to allow calculation of an average value. In the SEA trials, a venous blood sample was also collected via venepuncture from the antecubital vein in heparinized vacutainers and immediately spun in a centrifuge (Eppendorf AG, Hamburg, Germany) at 2500 rpm for 12 min at 4 °C. Plasma was transferred into epindorphs and stored at −80 °C until analysis. The venous sample was not collected in ALT as samples could not be stored or analysed in the mobile laboratory.

Following the pre-exercise measures, the participant mounted the ergometer to begin cycle exercise. The experimental protocol began with three x six minute stages of stationary cycling exercise at power outputs corresponding to 50, 65 and 80% of their previously determined VO_2max._ In the last minute of each stage, VO_2_ was measured as previously described. Perceived exertion (RPE) was also recorded using Borg G [34] Scale of Perceived Exertion. Then, following a five minute rest, the load on the ergometer was increased to a workload calculated to elicit VO_2max_ and participants were instructed to ride at this intensity, at a cadence of ≥ 80 rpm for as long as possible, with the trial being terminated once the participant could not maintain the required cadence (for the previous 10 s) or at volitional exhaustion. Time to fatigue at 100%VO_2max_ (TTE100%) was chosen in this study as a performance measure rather than a self-paced time trial to enable physiological data to be collected and compared at the five minute point. Accordingly, five minutes into the TTE100% VO_2_ was measured.

### Blood analysis

Plasma samples were analysed using a Nitric Oxide Colorimetric Assay Kit (BioVision Incorporated, Milpitas, California, USA) to measure NO_3_^−^ and NO_2_^−^. This method is a two-step process, in which nitrate reductase is used to convert NO_3_^−^ to NO_2_^−^, and then Greiss Reagents convert NO_2_^−^ to a deep purple azo compound. Absorbance is read at 540 nm and plotted as a function of NO_3_^−^ and NO_2_^−^ concentration.

### Statistical analyses

Statistical analyses to compare the values of all variables measured were done using statistical computer software (SPSS Statistics, Version 23, IBM Corporation, New York). Normal distribution of data was confirmed with the Shapiro-Wilks test. Variables measured during the submaximal exercise stages were analysed using three-way repeated measures ANOVA to test the significance level for main effects of, and interactions between, altitude (SEA or ALT), workload (50, 65 and 80%VO_2max_) and treatment (PLAC or POMx). Similarly, two-way (altitude x treatment) repeated measures ANOVA was performed on all variables measured during the TTE100%. Where significant interactions (*p* ≤ 0.05) were observed, two-tailed paired t-tests with a Fisher’s LSD post-hoc analysis were used to identify the location of the significance. The effect sizes (ES) of significant interactions were calculated using Cohen’s d (0.1 small; 0.5 medium; 0.8 large) [35]. The relationship between VO_2_ and performance during the TTE100% was analysed using the Pearson correlation coefficient. Data is presented as mean ± SD or mean change and 95% CI, as appropriate.

## Results

Participants’ self-reported adherence to supplement intake was 100% and no side effects of supplementation were reported. There was no order effect present for any of the significant results (*p* > 0.05).

### TTE100% performance

**Fig. 2:**
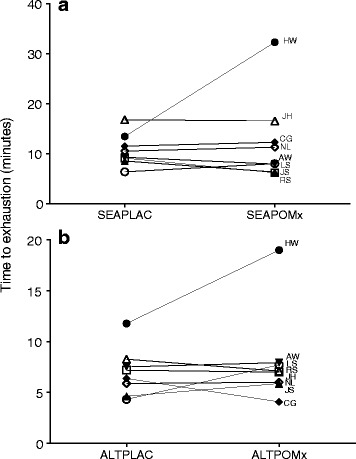
Individual TTE100% performance responses to POMx in (**a**) SEA and (**b**) ALT

Average performances during each condition were: SEAPLAC: 10.7 ± 2 min, SEAPOMx: 12.6 ± 8.7 min, ALTPLAC: 7.0 ± 2.3 min and ALTPOMx: 8.2 ± 4.5 min. There was no main effect of treatment on performance (*F*_1,7_ = 0.776, *p* > 0.05). Performance was significantly decreased at ALT compared to SEA (−4.1 min, 95% CI, −6.9, −1.3, *F*_1,7_ = 12.3, *p* < 0.02, ES = 0.8). The individual performance responses in the TTE100% to POMx in SEA and ALT are displayed in Fig. 2. Despite no overall significant effect of POMx, there appear to be two participants (HW and LS) who increased their performance with POMx in both altitudes.

### Resting measures

Plasma NO_3_^−^ was greater following POMx compared to PLAC (+10.3 μmol, 95% CI, 0.8, 19.7, *F*_1,7_ = 7.83, *p* < 0.04, ES = 0.9). However, the assay used was not sensitive enough to detect changes in NO_2_^−^. SBP was not significantly affected by POMx or ALT (*p* > 0.05). However, there was a trend towards an increase in SBP with POMx vs. PLAC (+3.9 bpm, 95% CI, −0.6, 8.5, *F*_1,7_ = 4.28, *p* = 0.08, ES = 0.3). There was a strong trend towards an increase in DBP with ALT, which showed a moderate effect size (+5 mmHg, *F*_1,7_ = 1.18, *p* = 0.054, ES = 0.6) and a significant treatment x altitude x time interaction (*F*_1,7_ = 7.64, *p* < 0.03). However, post-hoc analyses revealed no significant differences between pre or post-exercise DBP with either treatment at either altitude (*p* > 0.05). Hct was significantly increased by ALT (+1.4%95% CI, 0.2, 2.6, *F*_1,7_ = 7.56, *p* < 0.03, ES = 0.4) and significantly decreased by POMx, although the effect size was small (−0.76%, 95% CI, −1.3, −0.2, *F*_1,7_ = 10.4, *p* < 0.02, ES = 0.2).

### Pulmonary gas exchange, ventilatory and blood lactate responses to submaximal exercise

There was no significant main effect of POMx nor any significant altitude x treatment interaction on VO_2_, VCO_2_, HR or [La^−^]_b_ (*p* > 0.5). There was a trend towards an increase in HR with POMx, but this had a small effect size (+2.3 bpm, 95% CI, −5.0, 0.5, F_1,7_ = 3.80, *p* = 0.09, ES = 0.1). There was a significant main effect of altitude on [La^−^]_b_ (−0.8 μmol,95% CI, 0.3, 1.3, *F*_1,7_ = 2.95, *p* < 0.01, ES = 0.6).

### Pulmonary gas exchange, ventilatory and blood lactate responses to the time trial to exhaustion at 100%VO_2max_

The variables measured during the TTE100% are summarized in Table 1. All data was recorded five minutes into the TTE100%. The two-way ANOVA showed a significant altitude x treatment interaction for VO_2_ (*F*_1,7_ = 12.5, *p* = 0.01). Post-hoc analysis identified that POMx significantly increased VO_2_ at ALT (+3.8 ml.min^−1^kg^−1^, 95% CI, −5.7, 9.5, F_1,7_ = 29.2, *p* = 0.001) but not at SEA (F_1,7_ = 0.95, *p* > 0.05, ES = 0.2). Cohen’s d showed a moderate effect size of 0.6. There was no correlation between VO_2_ during the TTE and performance (*r* ^2^ = 0.0001, *p* ≥ 0.05). There was a significant altitude x treatment interaction for VCO_2_ (F_1,7_ = 6.32, *p* = < 0.04). However, post-hoc analysis identified no significant differences between treatments at SEA (F_1,7_ = 1.76, *p* > 0.5, ES = 0.2), and a trend towards an increase in VCO_2_ with POMx at ALT with a moderate effect size (+3.6, 95% CI, −0.665, 7.82, F_1,7_ = 3.98, *p* = 0.09, ES = 0.5). HR and [La^−^]_b_ were not affected by POMx or altitude (*p* > 0.5).

**Table 1:** Variables measured during the TTE100%

	SEA-PLAC	SEA-POMx	ALT-PLAC	ALT-POMx
VO_2_ (ml.min.kg^−1^)	70.0 ± 6.3	68.5 ± 7.2	63.2 ± 5.6^a^	66.9 ± 5.3^bc^
VCO_2_ (ml.min.kg^−1^)	72.4 ± 7.8	70.6 ± 8.9	69.6 ± 7.1	73.2 ± 8.0
HR (bpm)	186 ± 8	175 ± 8	184 ± 7	184 ± 5
[La^−^]_b_ (μmol.L^−1^)	11.0 ± 3.7	10.2 ± 2.9	12.2 ± 3.9	11.7 ± 3.9

Data are presented as means ± SD

*VO*_*2*_ oxygen uptake, *VCO*_*2*_ expired carbon dioxide, *HR* heart rate, *[La* ^*−*^ *]*_*b*_ blood lactate concentration, *SEA-PLAC* sea-level placebo, *SEA-POMx* sea-level pomegranate extract, *ALT-PLAC* high-altitude placebo, *ALT-POMx* high-altitude pomegranate extract

^a^indicates P < 0.05 compared to SEA-PLAC

^b^indicates *p* < 0.05 compared to SEA-POMx

^c^indicates *p* < 0.05 compared to ALT-PLAC

## Discussion

Although acute POMx supplementation was associated with a 10.3 μmol increase in plasma NO_3_^−^ (95% CI, 0.8, 19.7, *p* < 0.04, ES = 0.9), its use as an acutely-ingested ergogenic supplement by highly-trained athletes is not supported by the current study, as neither performance nor submaximal VO_2_ were significantly altered by POMx ingestion. However, our data indicated that POMx does allow maintenance of VO_2_ at a workload prescribed to elicit 100% VO_2max_ (at sea level) during high intensity exercise under low PO_2_ conditions, despite no significant performance effect.

The absence of changes in performance during the TTE100% is in agreement with Trinity et al. [32] who demonstrated no effect of acute POMJ supplementation on a cycling time trial or time to exhaustion protocol in hot conditions in moderately-trained individuals. However, Trexler et al. [7] found an increase in time to exhaustion during treadmill running at 90–100% of peak velocity, in moderately trained participants, following acute supplementation of POMx (1000 mg, 30 min before exercise) at sea-level. In addition, POMx has been shown to improve performance and recovery from resistance and sprint cycling exercise [36, 37]. The current study is the first study to test POMx supplementation in highly-trained endurance athletes and is in accordance with previous research involving acute BRJ supplementation in this cohort, which found no change in performance in a running or cycling time trial in hypoxic conditions [28, 29]. However, two studies involving multi-day periods of NO_3_^−^ supplementation in low PO_2_ conditions (11–13% O_2_) showed changes in both O_2_ parameters and exercise performance in moderately-trained individuals (VO_2max_ 58–61 ml.min^−1^kg^−1^). Kelly et al. [31] found that three days of BRJ supplementation (~8.4 mmol.day^−1^) decreased steady-state VO_2_ during a bout of moderate-intensity cycle exercise by 7.6% and this resulted in an 8% improvement in a subsequent high intensity time to exhaustion protocol, compared to exercise done following the intake of a placebo. Masschelein et al. [30] found that six days of BRJ supplementation (~5 mmol.day^−1^) increased arterial O_2_ saturation by 2.7% and the muscle tissue oxygenation index in the vastus lateralis by 4% during submaximal cycling. This resulted in a 5% increase in performance during a subsequent cycling graded exercise test. Thus, the small increase in VO_2_ with acute supplementation of POMx may not be sufficient to produce an ergogenic effect during a 100%TTE in highly-trained athletes and a longer period of supplementation may be necessary. Alternatively, the absence of change in performance despite an increase in VO_2_ may reflect the lack of correlation between VO_2_ and performance times (r^2^ = 0.0001), which indicates that VO_2_ was not the determining factor in the TTE100%. Rather, it is likely that other factors, such as anaerobic capacity, contribute more greatly to performance in a high-intensity TTE.

To the authors’ knowledge, the current research is the first study to have measured VO_2_ during cycling exercise following supplementation with POMx. The lack of effect of POMx on submaximal VO_2_ or VCO_2_ values in either environment despite an increase in plasma NO_3_^−^ is in accordance with previous research involving acute supplementation with NO_3_^−^-rich BRJ in highly-trained athletes (VO_2max_ >66 ml ^−1^min^−1^kg^−1^) conducted at sea-level [23, 24, 26, 38] or in low PO_2_ conditions (13–15% O_2_) [28, 29]. In addition, the absence of change in VO_2_ during the TTE100% at SEA correlated with Boorsma et al. [39] who conducted a running protocol involving similar intensities to the current study, and found no significant difference in VO_2_ values during a 1500 m TT. The restoration of an ALT-induced lowering in VO_2_ during intense exercise following POMx compared to SEA (+3.8 Lmin^−1^, 95% CI, −5.7, 9.5, *p* = 0.001, ES = 0.6) differed from previous NO_3_^−^-based research involving highly-trained participants, although no other studies using hypoxia and VO_2_ measurement have utilised a performance test protocol similar to ours. Masschelein et al. [30] found no change in VO_2max_ recorded during a graded exercise test to exhaustion in hypoxic conditions (11% O_2_, ~5000 m altitude) despite a 4% increase in the muscle tissue oxygenation index. However, in that study, exercise intensity was not stable, as it was in the current study and VO_2_ was measured at exhaustion, rather than part-way into the exercise. In addition, MacLeod et al. [29] recorded no change in VO_2_ during a 10 km cycling time trial completed in a normobaric hypoxic chamber (~2440 m altitude). However, the duration of this test was significantly longer than ours, which may have influenced potential effects on VO_2_.

The changes in VO_2_ in the current study were likely driven by the high polyphenol content of POMx, as POMx has a lower NO_3_^−^ concentration than BRJ. While BRJ-based research in highly-trained athletes has recorded increases in plasma NO_3_^−^ of 31–1907% [27, 31, 39], the current study measured a 44% increase in NO_3_^−^ with POMx. Although polyphenolic compounds are also present in BRJ, these are predominantly from the quercetin subclass, in comparison to the high ET component in POMx [40]. Previous research has generally found no link between quercetin intake, and changes in VO_2_ or performance parameters [41, 42]. However an increase in pre or post-exercise blood flow and vessel diameter has been observed with consumption of ET or anthocyanin-containing fruit juices and extracts [6, 7, 11]. Further, Ignarro et al. [17] demonstrated that POMJ is more effective at protecting NO from breakdown than other polyphenol-containing juices and red wine, with significant antioxidant actions occurring at dilutions greater than 1000-fold*.* Thus, the increased VO_2_ during the TTE100% following POMx supplementation may be due to a polyphenol-induced greater NO bioavailability.

Despite the large amount of research indicating a relationship between a supplement-induced increase in NO and changes in VO_2_ during exercise, presumably through an increase in the efficiency of O_2_ transport, the mechanisms behind these changes are currently irresolute. Although NO is known to mediate vasoactivity, an overall increase in vasodilation does not explain the reduction in VO_2_ during submaximal exercise in normoxia which has been observed in other studies [18–22]. Rather, the predominant mechanistic theories to explain the effects of NO on VO_2_ involve an increase in either the efficiency of mitochondrial O_2_ usage or in the muscular use of ATP, affording a lower VO_2_ requirement to sustain a given work rate [21, 43]. However, during exercise under hypoxic conditions, a NO-induced augmentation in vasodilation has been shown to be important in maintaining blood flow to the active muscles [44]. Thus, it is likely that under hypoxic conditions, the vasoactive role of NO contributes more greatly to the level of VO_2_ which can be achieved during intense exercise. In accordance with previous research [26, 28], POMx did not affect [La^−^]_b_, indicating that any changes observed were probably not due to a change in fuel usage.

Several reasons have been proposed for the lower efficacy of NO_3_^−^ supplementation on submaximal VO_2_ and performance in trained compared to untrained individuals. Firstly, as a response to training, athletes tend to have elevated NOS activity [45], and higher resting NO_2_^−^ and NO_3_^−^ values [46]. Consequently, they may have a lower requirement for NO_3_^−^-NO_2_^−^-NO pathway, and have sufficient NO_3_^−^ present in the blood to use it when needed. Further, training adaptations which aid in O_2_ transport and energy production, such as increased capillarization and mitochondrial density, may reduce the incidence of acidic and hypoxic muscular environments, which decrease NOS activity, and increase reliance on the NO_3_^−^ pathway [25, 47]. Finally, research in rats has suggested that the NO_3_^−^ pathway is predominantly used in type II muscle fibres, which work more frequently under acidic or hypoxic conditions [48]. If this is the case, NO_3_^−^ supplementation may be more effective in untrained individuals who tend to have a greater percentage of type II fibres [49]. However, currently, there is no direct evidence that type II fibres are preferentially affected in humans. Alternatively, the lack of overall performance response may be due to the presence of ‘responders’ and ‘non-responders’ to POMx. Despite no overall significant effect of POMx on performance in the current study, there were two participants who increased performance in both altitudes. Thus, a larger sample size of participants may be needed to determine the ratio of responders vs. non-responders in a highly-trained population.

In addition to the results already presented in the discussion, the current study produced two results which are difficult to explain. Firstly, in contrast to previous research which has reported a lowering in SBP following acute BRJ or ET polyphenols from grapes in sedentary or moderately-trained individuals [11, 18, 50], and no effect on SBP following BRJ supplementation in highly-trained individuals [25, 27, 29, 38], this study found a trend towards an increase in SBP with POMx (+3.9 bpm, 95% CI, −0.6, 8.5, *p* = 0.08). The only explanation we can give for this result is that the increase in SBP was a reactive response by the vasculatare to ensure that the mean arterial pressure and thus, vessel perfusion and blood flow, were maintained, despite the NO-induced vasodilation. Further, being an acute intervention, we did not anticipate changes in Hct with POMx, but surprisingly, there was a significant decrease in Hct following POMx compared to PLAC (−0.76%, 95% CI, −1.3, −0.2), which indicated a small reduction in the O_2_-carrying capacity of the blood. However, despite being significant, this change had a small effect size (ES = 0.2) and considering the increase in VO_2_ with POMx at ALT, did not appear to affect VO_2_ capacity. Hct varies from day to day by ~3% [51] and it is possible that this result was due to differences in hydration between tests, as we did not measure pre-exercise hydration status, or standardize water intake during exercise. However both these explanations are purely speculative and further research is required to determine whether POMx supplementation consistently results in similar effects on SBP and Hct.

A limitation to the current study is the relatively low altitude used in the hypoxic condition compared to the altitude generally associated with physiological changes (2500–3000 m) [52] and that simulated in previous studies involving NO_3_^−^ supplementation in hypoxia (~2500–5000 m) [28, 30, 31]. Due to our study being conducted in a mobile laboratory, we were restricted to areas with vehicle access in the North Island of New Zealand, and conducted the study at the highest altitude possible under these conditions. However, future studies could investigate the potential ergogenic benefit of POMx at a higher altitude of >2500 m. In addition, the current study was limited by a relatively small sample size (*n* = 8), which was unavoidable due to the research taking place during a high-performance junior training camp. While previous research has demonstrated a reduction in VO_2_ during submaximal cycling exercise [18, 21], it is acknowledged that the lack of effect of POMx on submaximal VO_2_ in the current study may have been due to an insufficient number of participants.

## Conclusion

In conclusion, acute POMx supplementation allowed a partial restoration of VO_2_ during intense exercise in a hypoxic environment. However, no significant changes in VO_2_ occurred during submaximal exercise and there was no effect of POMx on performance in either environment. Thus, the results from the current study do not support POMx as an ergogenic supplement when ingested acutely prior to exercise.

## Abbreviations

[La^−^]_b_: Plasma lactate concentration
ALT: High altitude conditions
BRJ: Beetroot juice
CO_2_: Carbon dioxide
DBP: Diastolic blood pressure
ET: Ellagitannin polyphenols
Hct: Haematocrit
HR: Heart rate
NO: Nitric oxide
NO_2_^−^: Nitrites
NO_3_^−^: Nitrates
NOS: Nitric oxide synthases
O_2_: Oxygen
PLAC: Placebo
POM: Pomegranate
POMJ: Pomegranate juice
POMx: Pomegranate extract
RER: Respiratory exchange ratio
ROS: Reactive oxygen species
RPE: Rating of perceived exertion
SBP: Systolic blood pressure
SEA: Sea level conditions
TTE100%: Time trial to exhaustion at 100%VO_2max_
VCO_2_: Volume of expired carbon dioxide
V_E_: Volume of expired air
V_I_: Volume of inspired air
VO_2_: Volume of oxygen consumption
VO_2max_: Maximal oxygen consumption
